# Efficacy of arginine depletion by ADI-PEG20 in an intracranial model of GBM

**DOI:** 10.1038/s41419-018-1195-4

**Published:** 2018-12-13

**Authors:** Justyna Magdalena Przystal, Nabil Hajji, Combiz Khozoie, Alexander Renziehausen, Qingyu Zeng, Fernando Abaitua, Amin Hajitou, Keittisak Suwan, Elizabeth Want, John Bomalaski, Peter Szlosarek, Kevin O’Neill, Tim Crook, Nelofer Syed

**Affiliations:** 1 Phage Therapy Group, Division of Brain Sciences, Imperial College London, London, UK; 2 John Fulcher Neuro-Oncology Laboratory, Division of Brain Sciences, Imperial College London, London, UK; 3 Department of Cancer and Surgery, Imperial College, London, UK; 4 Polaris Pharmaceuticals Inc., San Diego, California USA; 5 Center for Molecular Oncology, Barts Cancer Institute, Queen Mary University of London, Charterhouse Square, London, UK; 6 St Luke’s Cancer Centre, Egerton Road, Guildford, UK

## Abstract

Glioblastoma multiforme (GBM) remains a cancer with a poor prognosis and few effective therapeutic options. Successful medical management of GBM is limited by the restricted access of drugs to the central nervous system (CNS) caused by the blood brain barrier (BBB). We previously showed that a subset of GBM are arginine auxotrophic because of transcriptional silencing of *ASS1* and/or *ASL* and are sensitive to pegylated arginine deiminase (ADI-PEG20). However, it is unknown whether depletion of arginine in peripheral blood in vivo has therapeutic activity against intracranial disease. In the present work, we describe the efficacy of ADI-PEG20 in an intracranial model of human GBM in which tumour growth and regression are assessed in real time by measurement of luciferase activity. Animals bearing intracranial human GBM tumours of varying ASS status were treated with ADI-PEG20 alone or in combination with temozolomide and monitored for tumour growth and regression. Monotherapy ADI-PEG20 significantly reduces the intracranial growth of ASS1 negative GBM and extends survival of mice carrying ASS1 negative GBM without obvious toxicity. The combination of ADI-PEG20 with temozolomide (TMZ) demonstrates enhanced effects in both ASS1 negative and ASS1 positive backgrounds.Our data provide proof of principle for a therapeutic strategy for GBM using peripheral blood arginine depletion that does not require BBB passage of drug and is well tolerated. The ability of ADI-PEG20 to cytoreduce GBM and enhance the effects of temozolomide argues strongly for its early clinical evaluation in the treatment of GBM.

## Introduction

Glioblastoma multiforme (GBM) remains a disease in which, despite optimal management, prognosis is invariably poor with a median overall survival of 15 months. Effective medical management of GBM is compromised by the presence of the BBB which limits delivery of drugs into the central nervous system (CNS). TMZ is the most active cytotoxic agent in routine use for GBM but despite its clinical activity, particularly in patients with O6-Methylguanine-DNA-methyltransferase (MGMT) deficient disease, early relapse and death are common^1^.

We previously demonstrated that 30% of de novo GBM patients are auxotrophic for the amino acid arginine as a result of transcriptional silencing of *ASS1* and/or *ASL* which encode the two key enzymes in arginine biosynthesis, argininosuccinate synthetase (ASS1) and argininosuccinate lysase (ASL) respectively^2,3^. Arginine auxotrophy confers sensitivity to pharmacological depletion of arginine^4–11^ and this can be achieved using the drug pegylated arginine deiminase (ADI-PEG20) which catalyses the deimination of arginine and thereby depletes the level of arginine in biofluids. The concentration of arginine in cerebro spinal fluid (CSF) is estimated at 20–40 μM^12,13^ and is detectable in neurons and astrocytes. Arginine biosynthesis is very limited in the CNS and the majority of CNS arginine is derived from biosynthesis in the kidney and import from peripheral blood. Arginine enters the CNS via transport systems localised in the endothelial cells of the blood brain barrier (BBB) and the increased demand for arginine by cancer cells is met by extraction from blood rather than increased synthesis in the CNS^14^. Our previous work showed a strong correlation between arginine auxotrophy and sensitivity to ADI-PEG20 in both established GBM cell lines and primary cultures of GBM^3^. The most common mechanism of reduced expression of *ASS1* and *ASL* in GBM is transcriptional silencing mediated via CpG island methylation. The strong selective pressure for down-regulation of arginine biosynthesis is underlined by our observation of epigenetic silencing of both *ASS1* and *ASL* in a subset of cases with concomitant hypersensitivity to arginine deprivation. These in vitro data were of particular interest because they suggest a means of treating GBM that bypasses the requirement for passage across the BBB. Moreover, the correlation between *ASS1* and/or *ASL* silencing and sensitivity to ADI-PEG20 provides a relatively simple biomarker by which patients can be identified in whom this therapy may be appropriate. It is, however, unknown, whether depletion of arginine in peripheral blood translates into a meaningful cytostatic or cytoreductive response of GBM in vivo. Moreover, nothing is known of (possible) interaction(s) between arginine deprivation and sensitivity to TMZ. Resolution of these questions is important if ADI-PEG20 is to be considered as a candidate therapeutic for clinical GBM. To address these issues, we employ the use of an orthotopic model of GBM to assess the in vivo intracranial efficacy of ADI-PEG20 in GBM.

Our results have demonstrated that ADI-PEG20 efficiently depletes blood arginine and significantly reduces the growth of intracranial ASS1 negative GBM in mice extending their survival. Furthermore, combined treatment of ADI-PEG20 with temozolomide reduced the growth of ASS1 positive GBM.

Together, our results provide proof of principle for a therapeutic strategy for GBM that does not require BBB passage and suggest he combination of ADI-PEG20 with TMZ as a novel therapeutic strategy for both ASS1 negative and ASS1 positive GBM.

## Methods

### Ethical approval

This study was approved by Imperial College London Research and Ethics Committee (REC 14/EE/0024).

### Generation of transduced reporter GBM cell lines and their sensitivity to ADI-PEG20

All cell lines were purchased from ATCC and maintained in DMEM supplemented with 10% foetal bovine serum. To generate stable lines expressing GFP and luciferase, LN229 and U87 parental cell lines, (ASS1 negative and ASS1 positive respectively^3^,) were plated at a density of 5 × 10^4^ cells per well in 24 well plates. The next day when cells had reached 50–70% confluency the culture medium was removed and replaced with fresh medium containing Transdux TM (1x final concentration). Cells were then transduced with the lentivirus vector having dual expression of GFP and Luciferase under the control of a CMV promoter (Cambridge bioscience). A MOI of 100 was used for LN229 and 50 for U87. Plates were gently swirled to allow mixing of the contents. Expression of GFP was monitored by fluorescence microscopy and 9 days post transduction, GFP positive cells were sorted by FACS and expanded. Luciferase expression was measured using the Steady-glo® luciferase assay kit (Promega).

Sensitivity of transduced cell lines to ADI-PEG20 (Polaris Pharmaceuticals Inc., San Diego, CA, USA) was assessed as described previously^3^ using the sulphorodamine B (SRB) assay (Sigma-Aldrich). Essentially, parental and transduced cells were seeded in triplicate in 96-well plates at a density of 4 × 10^3^ cells per well. Twenty-four hoursafter seeding, the cells were washed three times in PBS and cultured in medium containing 1 μg/ml ADI-PEG20 supplemented with 1 mM citrulline and 2% dialysed FCS for various time points. Cells were fixed with 10% tri-chloroacetate (TCA) for at least 1 h at 4 ^o^C, washed with distilled water and allowed to dry before being stained with 0.4% SRB for 30 min at RT. The unbound SRB was washed with 0.1% acetic acid and the plates allowed to air dry. Bound SRB was dissolved in 150 μl of 10 mmol/l Tris pH 10.5 and absorbance read at 495 nm. These transduced lines were also tested in 3D culture together with a panel of patient derived primary GBM lines as follows: 5000 cells/well were seeded in low attachment 96 well plates (Thermo Scientific) in DMEM or DMEM/F12 medium supplemented with 10%FCS for transduced and primary cells respectively. After 3 days when spheres could be detected,1 μg/ml ADI-PEG20 was added and spheres allowed to grow for a further 2 weeks. Images were captured and sphere size measurements analysed using image J software.

**Generation of ASS1 knockdown and over-expressing cell lines**.

### *ASS1* shRNA knockdown

The target sequences for human ASS1 were 5′-GCCTGAATTCTACAACCGGTT-3′ (*ASS1*-shRNA-1) and 5′-ATGAACGTGCAGGGTGATTAT-3′ (*ASS1*-shRNA-2) and obtained from the RNAi Consortium^15^. These were individually inserted into an EcoRI and AgeI cut pLKO.1 - TRC cloning vector (Addgene plasmid # 10878) and validated by sequencing (Genewiz Inc., Cambridge, MA). Subsequently these plasmids were used for lentiviral particle production and target cell (TB48) transduction following the Addgene protocol for lentiviral transduction of mammalian cells (https://www.addgene.org/tools/protocols/plko/#A). The luciferase targeted shRNA was used as the non-silencing control. The sensitivities of these ASS1 knock down lines to ADI-PEG20 was carried out as described above.

### *ASS1* overexpression

The *ASS1* ORF was purchased from Origene and sub-cloned into the pBabe-puro vector (Addgene plasmid # 1764). Stable pBabe (vector control) and pBabe-puro-*ASS1* cell lines were established by retrovirus transduction as previously described (Pear et al. 1998). Briefly, each construct was co-transfected together with pUMVC and pCMV-VSV-G packaging and envelope plasmids into HEK293T/17 cells using polyethylenimine (PEI). Forty-eight hours after transfection, the supernatant containing retrovirus was filtered through a 0.45 µM filter, supplemented with 8 µg/mL of polybrene, and used to transduce target cells (LN229 and GBM31). Twenty-four hours after transduction, puromycin selection was applied for seven days to generate populations of stable transduced cells that overexpress *ASS1*. The sensitivities of these ASS1 over-expressing lines to ADI-PEG20 was carried out as described above.

### Measurement of serum arginine and citrulline

The levels of arginine and citrulline in serum was determined by liquid chromatography-tandem mass spectrometry (LC-MS/MS)^16,17^ following sampling of peripheral blood from the tail vein at various intervals as follows. In tumour free mice, blood was collected on 0, 5, 7, 12 and 14 days post primary injection of 5IU ADI-PEG20 or 0.9% saline intramuscularly (IM). In tumour bearing mice, blood was collected before surgery, three days after surgery and three days after each ADI-PEG20 or saline treatment for the duration of the experiment. Blood samples were incubated at 37 ^0^C for 30 min to allow clotting and serum separated by centrifugation at 10,000 × *g* and stored at −80 ^0^C until used. For analysis by LS-MS/MS, serum samples were thawed on ice and 5 μl mixed with 15 μl of methanol containing 0.1% (v/v) formic acid to remove proteins. Samples were then vortex and incubated at 20 ^0^C for 20 min before being centrifuged at 10,000 × *g* for 10 min. Five microlitres of sample was used for derivatization alongside 5 μl of amino acid standards as follows. Five microlitres of each standard, quality control (QC) or sample was added into a deep well (1 ml) of a 96-well plate (10 μl for the double blank). Five mirolitres of of Internal Standard (IStd) mix was added to each well (except the double blank) and 70 μl of borate buffer was added to each well. The plate was capped, vortex mixed gently and centrifuged for 2 min at 2000 *g*. Twenty microlitres of AccQTag reagent was added to each well, the plate was capped, and vortex mixed gently, left at RT for 1 min then placed on a heat block at 55° for 10 min. The plate was vortex mixed gently and diluted 1:99 (v/v) by adding 10 μl derivatised sample to 990 μl water. The samples were analysed using the conditions described in the previously published protocol^16^. Briefly, the amino acids were separated using an Acquity HSS column (2.1 × 150 mm, Waters, Milford USA) with a 5 min elution gradient of water and acetonitrile (0.1% formic acid). Derivatised amino acids were detected using optimized MRM transitions via a TQ-S tandem quadrupole mass spectrometer (Waters).

### Generation of an intracranial model of GBM

This project was approved by the ethics committee at Imperial College London. Stably transduced GBM cells at 80% confluency were trypsinised, washed with PBS and resuspended in 6 µl of saline before being injected stereotactically into the striatum of anesthetized immunodeficient CD−1 nude mice. 3.5 × 10^5^ and 2.5 × 10^5^ cells were used for LN229/GFP-Luc and U87/GFP-Luc respectively. In brief, mice were anesthetized by inhalation of 2% isoflurane and 98% O2 and a sagittal incision of 1 cm in length was made to expose the skull surface. The skull was punctured using a drill at the coordinates: 2 mm to the right of the bregma and 1 mm to the coronal suture which had been stereotactically established previously. Cells were slowly injected into this cavity over a 6 min period after which the scalp was sutured and mice monitored post-operatively until they become ambulant and retained normal activity.

### Bioluminescence imaging (BLI)

To monitor luciferase activity/expression, mice were anesthetized as described previously and injected subcutaneously with 150 mg/kg of D-luciferin (Gold Biotechnology) in the neck area. Five minute post D-luciferin injection, photonic emission was imaged using the in vivo Imaging System (IVIS 100). Mice were imaged twice per week and bioluminescent images were quantified as photons/sec and plotted against time in days.

### Histopathology

At the end of all experiments, animals were anesthetised and perfused with 1x cold PBS. Brain were harvested by either snap freezing on dry ice then stored at −80 ^o^C, or were fixed in formalin by slowly injecting 20 ml into mice after perfusion with PBS. Fixed brains were collected, stored in formalin for 2 days and then embedded in paraffin. Eight micrometre cryostat sections were cut and processed for hematoxylin and eosin (H&E) staining and examined under light microscopy. Immunohistochemical analysis of brain sections for Ki67 (Abcam) and LC3B (Cell Signaling) was performed as per the manufacturer’s instructions.

### Statistics

GraphPad Prism software (version 5.0) was used to perform statistical analyses. Data is presented as mean ± standard error of the mean (s.e.m.). *P* values were generated by one-way or two-way ANOVA.

## Results

### Generation of cell lines to assess the efficacy of ADI-PEG20 in an orthotopic model of GBM

**Fig. 1:**
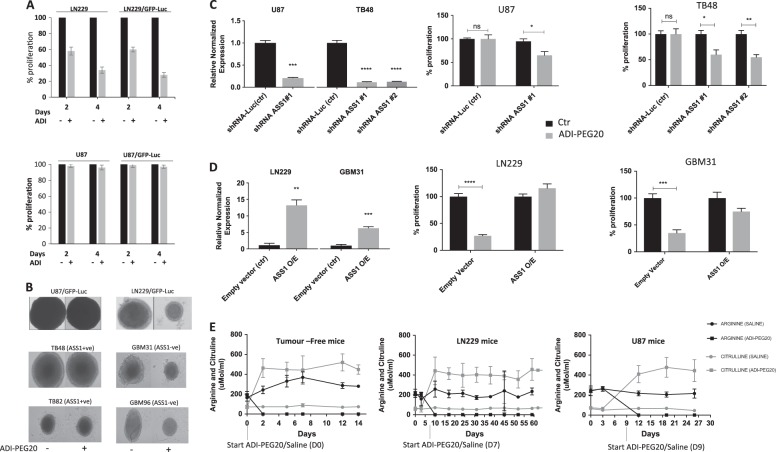
Parental and engineered GBM cells respond to arginine deprivation accordingly **a** GBM cells stably expressing GFP/Luc have comparable sensitivities to ADI-PEG20 as their parental counterparts. LN229 (upper panel) and U87 (lower panel) GBM cells stably expressing GFP/Luc (LN229/GFP-Luc and U87/GFP-Luc respectively) and their parental counterparts were plated into 96 well plates in triplicate and incubated overnight at 37 ^0^C. The next day, culture medium was replaced with fresh medium containing 1ug/ml ADI-PEG20 and 1 mM citrulline. Control wells received medium containing citrulline only. Cell proliferation was assessed on day 2 and 4 post ADI-PEG20 treatment using the SRB assay as described in Methods. Data shown are means +/− 1 SD of three experiments. **b** ADI-PEG20 inhibits the growth of ASS-ve cells in 3D. A panel of patient derived primary GBM lines were seeded in low attachment 96 well plates and allowed to form spheres.1 μg/ml ADI-PEG20 was added and spheres allowed to grow for a further 2 weeks. Sphere size measurements were analysed using image J software. **c** and **d** Knock-down of ASS1 confers sensitivity to ADI-PEG20 whereas over-expression confers resistance in both established and primary GBM cell lines. **e** ADI-PEG20 efficiently depletes serum arginine in tumour free mice and tumour bearing mice. Tumour free and tumour bearing mice were injected intramuscularly with 0.9% saline or 5IU ADI-PEG20. Blood collected from the tail vein at various time points was analysed for arginine and citrulline levels by LC-MS/MS as described in methods. Data shown are means +/−1 SD of six mice

We previously reported that arginine deprivation using ADI-PEG20 is effective at killing a subset of GBM cells in vitro due to methylation dependent transcriptional silencing of *ASS1*, the rate limiting enzyme of arginine biosynthesis^3^. To extend our findings to an orthotopic mouse model we first engineered LN229 (ASS negative) and U87 (ASS1 positive) GBM cells to constitutively express GFP and luciferase. Cells were transduced with lentivirus carrying a fusion of both genes as described in methods. Nine days post-transduction, GFP positive cells were sorted and allowed to grow before being assessed for luciferase activity and GFP reporter expression (Supplementary Figure S1A). Prior to in vivo experiments, transduced cells (LN229/GFP-Luc and U87/GFP-Luc) and their parental counterparts were treated with 1 µg/ml ADI-PEG20 and analysed for cell proliferation using SRB staining on days 2 and 4 post treatment. Transduced cells displayed sensitivities to ADI-PEG20 comparable to their parental counterparts (Fig. 1a upper panel and lower panel). We also tested these transduced cells in 3D cell culture together with a panel of patient derived primary GBM cells (Fig. 1b). As expected, ADI-PEG20 reduced neurosphere growth only of ASS-ve lines (LN229/GFP-luc, GBM31 and GBM 96). To further validate the hypothesis that absence of ASS expression correlates with response to ADI-PEG20, we used shRNA technology to knock down *ASS1* in ASS + ve U87-MG and TB48 cells and over express *ASS1* in ASS-ve LN229 and GBM31 cells. Knock down of ASS1 sensitized cells to ADI-PEG20 whereas over-expression rendered them resistant to treatment (Figs. 1c, d).

### ADI-PEG20 efficiently depletes blood arginine

Prior to testing the efficacy of ADI-PEG20 in our intracranial GBM model, we first determined the ability of the drug to lower serum arginine levels in tumour free CD−1 nude mice. Groups of 7mice were IM injected on day 0 and 7 with either 5IU of ADI-PEG20 or 0.9% saline and blood was collected by tail vein bleeding at various time points. Serum arginine and citrulline levels were determined by LC-MS/MS as described in methods. ADI-PEG20 reduced circulating arginine levels within 48 h and levels remained low for the duration of the study after a further dosing at day 7. As expected, there was a corresponding increase in citrulline, the breakdown product of arginine in these animals (Fig. 1e). In contrast, no reduction in the levels of arginine or increase in citrulline could be detected in the control saline treated group (Fig. 1e). However, we did observe an increase in the levels of arginine in this group which appeared to plateau at day 12. All animals remained healthy.

### ADI-PEG20 efficiently depletes blood arginine in tumour bearing mice but inhibits the growth of ASS1 negative tumours only

Having established an in vivo reporter system and observed efficient depletion of serum arginine in mice, we next proceeded to test the effect of ADI-PEG20 on the intracranial growth of ASS1 negative (LN229/GFP-*Luc)* and ASS1 positive (U87/GFP-Luc) GBM cells. Intracranial tumours were initiated by injecting exponentially growing cells into the striatum of CD−1 nude mice and monitored by BLI for luciferase activity twice/week for the duration of the study. Animals received ADI-PEG20 or saline on a weekly basis and blood was collected as described previously for analysis of arginine and citrulline levels.

**Fig. 2:**
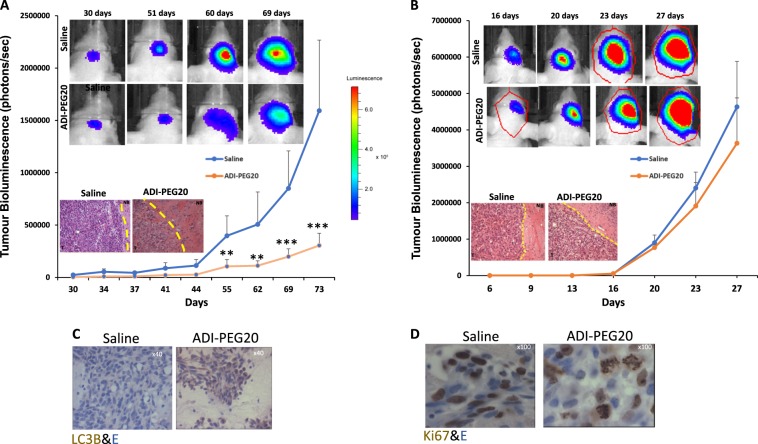
In vivo assessment of ADI-PEG20 in mice bearing ASS1 negative and positive GBM tumour. **a**, **b** CD−1 nude mice were stereotactically injected with LN229/ GFP-*Luc* or U87/ GFP-*Luc* cells as described in methods. On day 7 and 9 post injection of cells (first detection of luciferase activity by BLI for LN229/GFP-Luc and U87-GFP/Luc cells respectively) animals were injected IM with 0.9% saline or 5IU ADI-PEG20. Treatments were repeated on a weekly basis for the duration of the study and tumour growth assessed by BLI analysis twice per week. Bioluminescent images of representative animals are shown starting from day 30 onwards for animals bearing LN229 tumours and from day 16 onwards for mice bearing U87 tumours. Colour depicts relative luciferase signal. Bioluminescence signal plotted as photons/sec against time in days. *P* values: **P < 0.01, ***P < 0.001. The error bars are +/− 1 SD from seven animals. **a**, **b** Inset: H&E staining of representative tumour sections (x 1.25). T tumour, NB normal brain. **c**, **d** IHC analysis for LC3B and Ki67 in tumour sections from ADI-PEG20 and saline treated animals

The luciferase signal in animals injected with LN229/GFP-Luc cells remained low in both treatment groups until day 30 post injection of cells after which a steady increase in signal was detected in the saline group which continued to increase to day 73 (Fig. 2a). In contrast, the luciferase signal remained low for 44 days in the ADI-PEG20 treated animals and then a gradual increase was detected. This increase in luciferase signal was significantly lower than in the saline treated animals (Fig. 2a). All animals were sacrificed on day 74, the time point at which control animals exhibited signs of distress.

The luciferase signal in mice bearing U87/GFP-Luc cells remained stable in both treatment groups until day 16 and an increase was observed in both groups which continued to day 27. Animals were sacrificed on day 29 (Fig. 2b). Efficient depletion of blood arginine was detected in the serum of both of these experimental groups of animals treated with ADI-PEG20 but only ASS1 negative GBM (LN229/GFP-Luc) were sensitive to this depletion (Fig. 1e).

To further characterise the effects of ADI-PEG20 on the growth of these intracranial tumours the brains of mice were removed, frozen at −80 ^o^C or fixed in formalin and sectioned before being stained with H&E. ADI-PEG20 reduced the growth of ASS1 negative tumours (Fig. 2a inset) but had no effect on the growth of ASS1 positive tumours (Fig. 2b inset). Since nutrient starvation slows the cycling of cells and induces autophagy (previously demonstrated in vitro in Syed et al. 2013) we investigated the in vivo effects of ADI-PEG20 by IHC for Ki67, a marker of cell proliferation and LC3B, the most widely used marker to monitor autophagy. Ki67 staining could be detected in both treated and untreated sections but the intensity of staining was greater in the absence of ADI-PEG20 (Fig. 2d). LC3B was only detected in the presence of ADI-PEG20 (Fig. 2c). Our results suggests that ADI-PEG20 decreases cell proliferation and initiates autophagy in vivo.

Weight of mice bearing LN229 tumours remained stable in both treatment groups until day 74 when a sharp drop was observed in the saline group. In mice bearing U87 tumours, the weight remained stable in both groups until day 20 and then a drastic drop was observed thereafter (Supplementary Figure: S2A and B).

### Regression of an ASS1 negative intracranial GBM tumour by ADI-PEG20

**Fig. 3:**
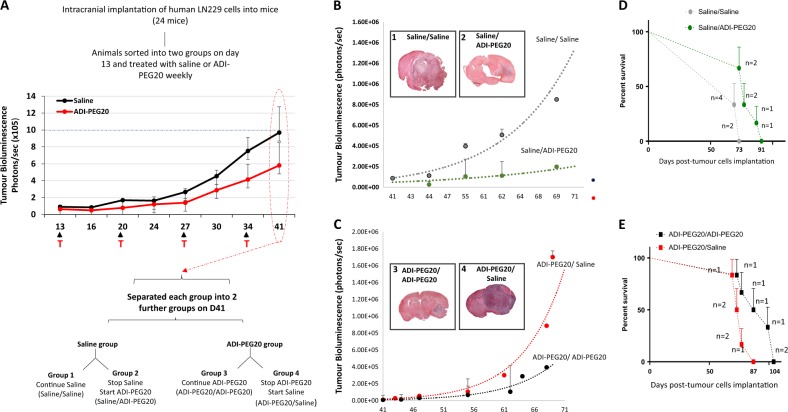
Assessment of tumour regression by ADI-PEG20. **a** Schematic representation of experimental setup to assess the efficacy of ADI-PEG20 on tumour regression in mice bearing ASS negative tumours. **b**,**c** Bioluminescence signals in mice plotted as photons/sec against time in days in each of the treatment groups. Group1: Saline/Saline; Group2: Saline/ADI-PEG20; Group3: ADI-PEG20/ADI-PEG20; Group4: ADI-PEG20/Saline. Error bars are +/−1 SD from six animals. Inset: H&E staining of representative brain sections from each treatment group after termination of the study on day 74. **d**, **e** Kaplein Meir survival curves

To determine if arginine deprivation could bring about regression of an established tumour and to assess if continued treatment was necessary for tumour suppression, we treated a cohort of animals with either saline or ADI-PEG20 until a clear difference in luciferase signals could be detected between the two groups and animals remained healthy (summarized in Fig. 3a). At this time point, ADI-PEG20 treatment was initiated in a cohort of animals in the saline group allowing for the assessment of tumour regression. Similarly, in the ADI-PEG20 group, treatment was stopped in a cohort to assess if continued treatment was necessary for tumour suppression.

Animals receiving continued ADI-PEG20 (group 3) or with delayed treatment (group 2) had substantially reduced tumour growth compared to those treated continuously with saline (group 1) or where ADI-PEG20 treatment was stopped (group 4) (Fig. 3b, c). ADI-PEG20 extended survival, with animals receiving continuous treatment surviving the longest (Fig. 3d, e).

### ADI-PEG20 potentiates the effects of Temozolomide in GBM irrespective of ASS1 status

**Fig. 4:**
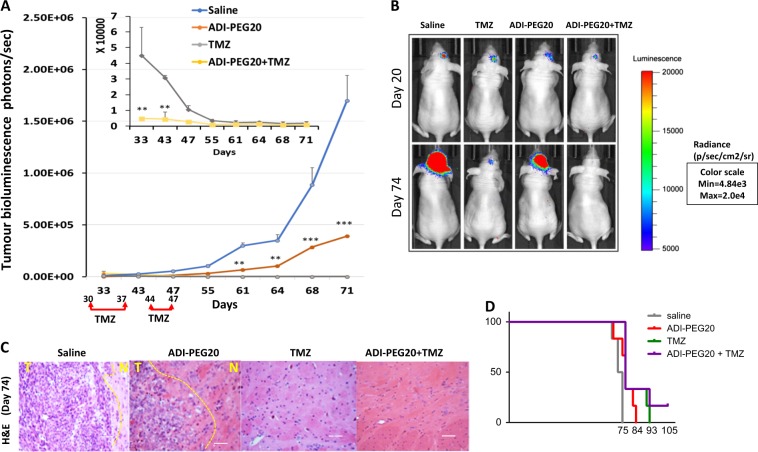
Assessment of therapeutic efficacy of combined treatment ADI-PEG20 and temozolomide in ASS1 negative GBM. **a**, **b** CD−1 nude mice were injected as described previously with LN229/GFP-Luc (24mice) GBM cells. Thirteen day’s post implantation mice were separated into 2 equal groups and each group received either IM injection of 5IU ADI-PEG20 or 0.9% saline weekly. On day 30 post implantation of cells each group was further divided into two groups. Animals in one group received TMZ (30 kg/ml) for 7 continuous days followed by a break of 7 days and received TMZ again for a further 4 days. ADI-PEG20 or saline treatment was continued on a weekly basis for the duration of the study. Representative images of bioluminescence signals in mice are indicated (**b**) and plotted as photons/sec against time in days (**a**) for each treatment group. **c** H&E staining of tumour sections **d** Kaplein Meir survival curve for combination treatment

**Fig. 5:**
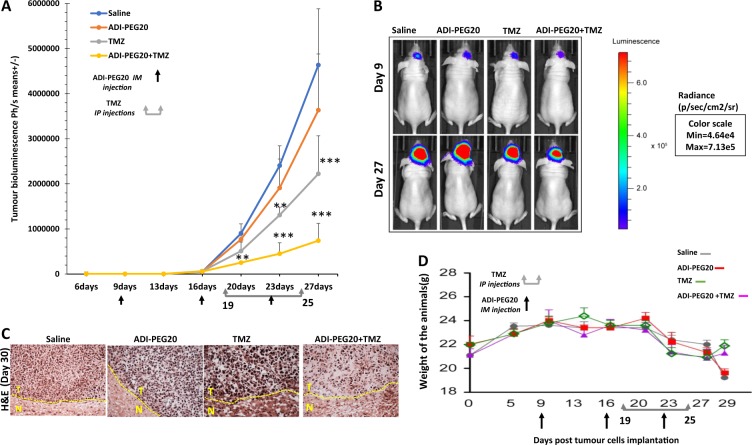
ADI-PEG20 demonstrates in vivo therapeutic synergy with temozolomide in ASS1 positive GBM. **a**, **b** CD−1 nude mice were injected as described previously with U87/GFP-Luc (24 mice) GBM cells. Nine +day’s post implantation mice were separated into two equal groups and each group received either IM injection of 5IU ADI-PEG20 or 0.9% saline weekly. On day 19 post implantation of cells each group was further divided into two groups. Animals in one group received TMZ (30 kg/ml) for 7 continuous days and then treatment was stopped. ADI-PEG20 or saline treatment was continued on a weekly basis for the duration of the study. Representative images of bioluminescence signals in mice are indicated (**b**) and quantified as photons/sec against time in days (**a**) for each treatment group. **c** H&E staining of tumour sections **d** Weight of animals during the course of the experiment. *P* values: **P < 0.01, ***P < 0.001. The error bars are +/− 1 SD from seven animals

Evidence in mesothelioma and other cancers implies that ADI-PEG20 may be more effective in combination with chemotherapy than as monotherapy^10,18,19^. We therefore proceeded to investigate the effects of combining ADI-PEG20 with TMZ, the current standard of care for GBM treatment. Mice were pre-treated with ADI-PEG20 or saline and then treated with TMZ as outlined in Figs. 4 and 5 and in Methods. As expected TMZ alone drastically reduced tumour growth in animals bearing LN229 tumours which are methylated for MGMT and the combination further reduced luciferase activity to undetectable levels (Fig. 4a, b). In contrast, TMZ had no significant effects in animals bearing U87 tumours which are unmethylated for MGMT but the combination significantly reduced luciferase activity in these ASS1 positive mice (Fig. 5a, b). Although these animals experienced dramatic weight loss from day 20 onwards, those receiving TMZ or combined ADI-PEG20 treatment recovered their weight by day 29 whereas animals receiving saline or ADI-PEG20 alone continued to lose weight (Fig. 5d). Only the saline treated ASS1 negative animals lost weight from day 69 onwards (Supplementary Figure S2). TMZ alone and in combination with ADI-PEG20 produced disruption of tumour architecture in ASS negative mice whereas in ASS1 positive mice this feature was only observed in the combined treatment group (Figs. 4c and 5c). Combined treatment extended the survival of mice with ASS negative tumours (Fig. 4d), whereas mice with ASS positive tumours were sacrificed on day 29 for humane reasons.

## Discussion

Despite the proven clinical activity of TMZ and emerging therapeutic options with immunotherapy and targeted agents, long term survival in patients with GBM is rarely observed^20^. As such, this is an area of high unmet clinical need in brain tumour management and additional novel therapeutic strategies are urgently required. A major restriction to the effective medical management of GBM is the limited passage of drugs across the BBB. In the present study, we have used an orthotopic model of GBM to assess the in vivo efficacy of the arginine-depleting agent ADI-PEG20.

Previous work, including our own, has demonstrated that arginine depletion is effective against ASS1 negative GBM cell lines, spheroids and xenografts^3,21^. Further, we showed that potent in vitro activity of ADI-PEG20 in arginine auxotrophic GBM cell lines and primary cultures correlates with methylation-dependent transcriptional silencing of *ASS1* and/or *ASL*^3,4^. Several mechanisms of action have been proposed as to how ADI-PEG20 induces cancer cell death. For instance, the duration of starvation by arginine depletion is thought to affect drug sensitivity of tumour cells. Moreover, ADI-PEG20 has been reported to be anti-angiogenic, alter the immune surveillance of tumour cells and induce autophagy^4,7,8,21–24^. The ability of ADI-PEG20 to efficiently deplete peripheral blood arginine suggests that its efficacy may not require BBB passage but there is no published evidence supporting this hypothesis. The goal of our study was to evaluate the candidacy of ADI-PEG20 in an in vivo model of GBM which resembles as closely as possible the clinical situation. In such a model system, we now show that depletion of blood arginine with ADI-PEG20 as monotherapy effectively treats ASS1 negative GBM and significantly extends the survival of mice bearing intracranial GBM xenografts. Moreover, in the same model we demonstrate that ADI-PEG20 potentiates the anti-GBM activity of TMZ. There are precedents for the concept of controlling intracranial cancer cell growth by targeting biochemical pathways outside the CNS and thereby avoiding the requirement for passage across the BBB. For example, in oestrogen receptor positive metastatic breast cancer, aromatase inhibitors such as letrozole and exemestane have activity against CNS metastatic disease by their inhibition of aromatase (which catalyses the conversion of androgens into oestrogen) and is expressed in peripheral fat but not in the CNS. Reduction in blood oestrogen from inhibition of aromatase with reduction of oestrogen-dependent cancer cell growth and clinical response^25^ has clear mechanistic parallels to the reduction in peripheral blood arginine and inhibition of CNS cancer growth achieved by ADI-PEG20.

In initial experiments, we generated stable cell lines expressing GFP and luciferase using adenoviral transduction of parental LN229 (ASS1 negative) and U87 (ASS1 positive) cells and confirmed that the sensitivities to ADI-PEG20 of the derivative cell lines exactly match those of the parental cells and is consistent with our previous work^3^. Next, we confirmed the ability of ADI-PEG20 to efficiently deplete serum arginine in the mouse host and observed a rapid reduction to undetectable levels. After a single dose of ADI-PEG20, levels of serum arginine began to rise after 7 days and this informed the dosing schedule for subsequent studies. We then proceeded to test the effect of ADI-PEG20 on the growth of intracranial GBM and we demonstrated that mice bearing ASS1 negative LN229/GFP-Luc cells showed greatly reduced growth relative to controls when challenged with ADI-PEG20. Previous work has reported that arginine depletion inhibits the growth of GBM xenografts^21^ but to the best of our knowledge the present results are the first demonstration that depletion of peripheral blood arginine is effective in treating intracranial GBM in the presence of an intact BBB. Although the BBB is disrupted in GBM, the magnitude of this disruption is thought not be sufficient enough to allow drug penetration in meaningful quantities^26^. Our data is therefore proof of concept that depletion of peripheral blood arginine is an effective treatment for GBM and bypasses the limitations to effective disease control imposed by the presence of the BBB.

ADI-PEG20 shows definite clinical activity as a monotherapy^18,27,28^. However, there are reports that the activity of ADI-PEG20 may be increased by combination with other agents^19,29,30^ and this prompted us to test in our intracranial model the effect of ADI-PEG20 and TMZ. Although TMZ monotherapy was effective, BLI remained positive and a residuum of GBM was present at the close of the experiment. In animals treated with ADI-PEG20 and TMZ, BLI remained completely negative and critically, histopathology revealed no evidence of residual GBM in the brains of animals treated with the combination. Moreover, survival of animals was maximal in the group treated with combination therapy. Of particular interest, we also observed therapeutic synergy between ADI-PEG20 and TMZ in ASS1 positive GBM. The combination produced a highly significant reduction in intracranial growth of U87 cells whereas ADI-PEG20 monotherapy had only a minor effect. The mechanism of this effect remains unclear, but a sensitization to radiotherapy by arginine deprivation has been described^31^. These results imply that ADI-PEG20 may be clinically useful in combination with TMZ in both ASS1 negative and ASS1 positive settings. They also suggest the possibility that other arginine auxotrophic tumours with intracranial metastatic disease may be amenable to medical management with ADI-PEG20 and CNS penetrating chemotherapy.

In conclusion, we report that using an orthotopic intracranial model of GBM, ADI-PEG20 as monotherapy and in particular, in combination with TMZ is a safe and highly active approach to therapy. Despite trials of numerous new agents, there has been no improvement in time-dependent outcomes in GBM since the positive results obtained with TMZ. As such, the clear anti-GBM efficacy of ADI-PEG20 in an intracranial disease model suggests a novel approach to treating GBM. A major factor to facilitate early clinical assessment of ADI-PEG20 is that the toxicity profile has been well established in other cancer types in which the drug has been shown to be well-tolerated and safe when combined with chemotherapy^19,29,32^. Based on the results we present in this study and the established safety and tolerability of ADI-PEG20, we believe that early clinical evaluation of the combination of ADI-PEG20 and TMZ in GBM is warranted.

## Electronic supplementary material

Supplementary data
