# Supplementary material for: Efficacy of arginine depletion by ADI-PEG20 in an intracranial model of GBM
Source: Cell Death Dis. 2018 Dec 13;9(12):1192. doi: 10.1038/s41419-018-1195-4 (PMC6294248; doi:10.1038/s41419-018-1195-4)
Supplement: Supplementary file 1 — Supplementary data [file 41419_2018_1195_MOESM1_ESM.docx]

**Supplementary Figures**

**Figure: S1**

**Generation of GBM cell lines stably expressing luciferase and GFP.**

A: LN229 and U87 GBM cells were transduced with lentivirus carrying double fusion eGFP and firefly luciferase reporter genes under the transcriptional control of the CMV promoter at a multiplicity of infection of 100. B: One-week post-transduction GFP positive cells were sorted using a fluorescence activated cell sorter and expanded. C: Evaluation of luciferase activity in GFP positive stable clones.

**Figure: S2**

**Assessment of animal weight**.

A-C: Average weight animals in each treatment group (Saline or ADI-PEG20, TMZ, ADI-PEG20 + TMZ) in animals bearing U87 and LN229 tumours. The error bars are +/- 1SD from 6 animals.
